# Exploring risk factors for new-onset atrial fibrillation in elderly patients with myocardial infarction based on serum NT-proBNP, RDW, Hcy, and clinical parameters: an exploratory study using LASSO-logistic regression

**DOI:** 10.3389/fcvm.2026.1744533

**Published:** 2026-04-30

**Authors:** Qun Fan, Yang Ling, Cong Fu, Ling Jiang, Jichun Liu, Xianghai Wang, Hao Yang

**Affiliations:** Department of Cardiovascular Medicine, The Affiliated Hospital of the Wannan Medical College, Wuhu, China

**Keywords:** acute myocardial infarction, atrial fibrillation, homocysteine, n-terminal pro-brain natriuretic peptide, red cell distribution width

## Abstract

**Objective:**

To investigate the association of serum N-terminal pro-brain natriuretic peptide (NT-proBNP), red cell distribution width (RDW), homocysteine (Hcy), and clinical parameters with new-onset atrial fibrillation (NOAF) in elderly patients with acute myocardial infarction (AMI), and to identify potential risk factors in an exploratory analysis.

**Methods:**

This retrospective exploratory study enrolled 204 elderly AMI patients (January 2020—December 2022), divided into NOAF (*n* = 41) and non-NOAF (*n* = 163) groups. Clinical and laboratory data were collected. LASSO regression was used for initial feature selection from candidate variables. Multivariate logistic regression was then employed to identify factors associated with NOAF. A nomogram was constructed to visualize the risk factor model. Given the exploratory nature and limited number of events (*n* = 41), model performance metrics including area under the ROC curve (AUC), calibration, and decision curve analysis were evaluated with caution, accompanied by internal validation using 1,000 bootstrap resamples to assess overfitting.

**Results:**

Significant differences were found between the NOAF and non-NOAF groups in gender, smoking status, hospitalization duration, Killip class, NT-proBNP, RDW, and Hcy (*P* < 0.05). LASSO regression identified six key predictors: gender, hospitalization duration, Killip class, NT-proBNP, RDW, and Hcy. Multivariate logistic regression confirmed these as independent risk factors: female gender (OR = 3.367, 95% CI: 1.193–9.498), longer hospitalization duration (OR = 1.110 per day, 95% CI: 1.021–1.206), Killip class II-IV (OR = 3.171, 95% CI: 1.157–8.689), higher NT-proBNP (OR = 4.677 per ng/mL increase, 95% CI: 1.774–12.328), higher RDW (OR = 1.358 per % increase, 95% CI: 1.163–1.585), and higher Hcy (OR = 1.814 per µmol/L increase, 95% CI: 1.414–2.328); all *P* < 0.05. The model showed good fit (Hosmer-Lemeshow *χ*² = 6.957, *P* = 0.541). The final combined nomogram model achieved an AUC of 0.926 (95% CI: 0.880–0.973) in the development set. Internal validation via 1,000 bootstrap resamples yielded an optimism-corrected AUC of 0.901. Calibration was assessed visually and statistically (Hosmer–Lemeshow *χ*² = 6.957, *P* = 0.541), showing good agreement between predicted and observed risks (Figure 4). Internal validation indicated good calibration (Cox-Snell R² = 0.593, Nagelkerke R² = 0.376), and decision curve analysis confirmed a high net clinical benefit.

**Conclusion:**

This exploratory study identifies gender, hospitalization duration, Killip class, NT-proBNP, RDW, and Hcy as factors significantly associated with NOAF in elderly AMI patients. However, due to the modest sample size, low events-per-variable ratio, and lack of external validation, these findings should be considered hypothesis-generating. The proposed model is not ready for clinical application. Further validation in larger, prospective cohorts is essential to confirm these associations and to develop a robust predictive tool.

## 1 Introduction

Acute myocardial infarction (AMI) refers to myocardial necrosis caused by acute, persistent ischemic hypoxia of the coronary arteries. As a severe form of coronary atherosclerotic heart disease, it is characterized by sudden onset, severe condition, and high mortality rate, ranking among the primary causes of death in middle-aged and elderly individuals (1–3). With the accelerating aging of the global population and the widespread prevalence of risk factors such as hypertension and diabetes, the disease burden of AMI continues to grow worldwide. Among patients, the elderly present the most significant diagnostic and therapeutic challenges due to their unique pathophysiological characteristics and complex clinical conditions (4). Previous data indicate that preventing and managing various complications during the acute phase and long-term recovery of AMI is crucial for improving prognosis. Among these, new-onset atrial fibrillation (NOAF) following AMI is the most common and severely harmful arrhythmic complication (5). Yndigegn T et al. (6) indicate that NOAF is not merely an isolated electrophysiological event. Its occurrence signals a dramatic deterioration in clinical outcomes, leading to hemodynamic instability, increased risk of left ventricular thrombosis and systemic embolism, exacerbated heart failure, significantly prolonged hospital stays, and ultimately an independent increase in all-cause mortality. Therefore, accurately identifying elderly AMI patients at high risk for future NOAF during early hospitalization provides a valuable “window of opportunity” for risk stratification, targeted monitoring, and early intervention. This is crucial for improving long-term patient outcomes and optimizing healthcare resource allocation.

Currently, prediction of NOAF in clinical practice largely relies on traditional clinical parameters such as age and infarct location. However, these indicators may be subjective or merely reflect macroscopic outcomes following myocardial injury, proving insufficient for elucidating the complex microscopic pathological mechanisms underlying NOAF (7). Therefore, identifying novel serum biomarkers that objectively and conveniently reflect these underlying mechanisms, and integrating them with traditional clinical parameters, represents a crucial direction for optimizing predictive models. Against this backdrop, serum biomarkers demonstrate significant potential. N-terminal pro-brain natriuretic peptide (NT-proBNP) serves as a sensitive indicator of elevated ventricular wall tension and volume overload, reflecting not only myocardial injury severity and heart failure risk but also acting as a crucial substrate for the onset and maintenance of atrial fibrillation (8–10). Meanwhile, Altieri C et al. (11) propose that red cell distribution width (RDW)-a parameter measuring red blood cell volume heterogeneity in routine blood counts-is a long-underestimated marker of systemic inflammation and oxidative stress. RDW is closely associated with promoting atrial electrical and structural remodeling, thereby triggering atrial fibrillation. Homocysteine (Hcy), an amino acid closely associated with atherosclerosis, contributes to the progression of coronary artery disease and atrial myocardial instability through multiple pathways. It induces endothelial dysfunction, promotes oxidative stress, and enhances thrombogenicity, thereby creating favorable conditions for the development of NOAF (12, 13). Although previous studies have separately examined the associations between NT-proBNP, RDW, or Hcy and AMI prognosis or atrial fibrillation, few reports have focused on predicting NOAF in elderly AMI patients by combining these three indicators with clinical characteristics. Furthermore, traditional logistic regression faces challenges in model stability and predictive efficacy when dealing with numerous candidate variables that may exhibit multicollinearity.

Therefore, this study aims to conduct a retrospective analysis of clinical and serological data from elderly AMI patients. By employing the modern statistical method of LASSO-Logistic regression, we will explore key variables from numerous candidate predictors and ultimately construct a nomogram model to visualize these risk associations. Given the exploratory nature and sample size limitations, the primary goal is to identify potential risk factors for NOAF, rather than to validate a definitive predictive model. The findings are intended to generate hypotheses and inform the design of larger, confirmatory studies.

## 2 Materials and methods

### 2.1 Ethical statement

This study was approved by the institutional review board and ethics committee. Given its retrospective nature and use of de-identified patient data, informed consent was not required as no risk or detriment to patients was anticipated. This exemption complies with regulations and ethical guidelines pertaining to retrospective research.

### 2.2 Study design

This retrospective study enrolled 204 elderly patients with acute myocardial infarction (AMI) treated at our hospital between January 2020 and December 2022. Participants were categorized into a non-orthostatic arrhythmia (NOAF) group and a non-NOAF group based on the presence or absence of NOAF documented in the medical record system.

### 2.3 Inclusion criteria

Inclusion criteria: (1) Meets the diagnostic criteria for AMI in the “Guidelines for the Management of Acute Myocardial Infarction” (14), with positive myocardial necrosis markers (troponin) or elevated CKMB levels exceeding twice the upper limit of normal; patients exhibit symptoms of myocardial ischemia and show ischemic changes on electrocardiogram; (2) Age ≥60 years; (3) Complete demographic and clinical data; (4) Chest pain onset <24 h prior to admission; (5) No cognitive impairment, with normal communication ability.

Exclusion Criteria: (1) History of atrial fibrillation; (2) Concurrent structural heart disease or heart failure; (3) History of cardiac surgery; (4) Severe hepatic or renal dysfunction; (5) Hematologic disorders; (6) Thyroid disease; (7) Acute or chronic inflammatory diseases; (8) History of stroke or surgery within 3 months prior to enrollment.

### 2.4 General data collection

Clinical data were collected via the electronic medical record system, including age, gender (male/female), body mass index, marital status (married/other), educational attainment (junior high school or below/high school or above), smoking history (smoking >1 cigarette/day for >1 year) (yes/no), drinking history (drinking >1 alcoholic drink/day for >1 year) (yes/no), concurrent diabetes [meeting criteria in Application of the Chinese Expert Consensus on Diabetes Classification in Clinical Practice (15)] (yes/no), concurrent hypertension [meeting diagnostic criteria in The Japanese Society of Hypertension Guidelines for Self-monitoring of Blood Pressure at Home (Second Edition) (16)] (yes/no), Admission heart rate, length of hospital stay, systolic blood pressure, diastolic blood pressure, infarct location (anterior wall/inferior wall/combined wall/other), number of diseased vessels (1 vessel/>1 vessel), Killip class of cardiac function (Class I/Class II–IV), target vessel (left main coronary artery/left anterior descending artery/circumflex artery/right coronary artery).

### 2.5 Laboratory parameters

Collect 5 mL of patient venous blood in the morning on an empty stomach. Centrifuge using a centrifuge (Model: LL900, Manufacturer: Luoyang Hongshi Machinery Equipment Co., Ltd.) at 3,000 rpm for 10 min to separate serum. Detect NT-proBNP using an enzyme-linked immunosorbent assay (ELISA). Detect interleukin (IL)-6 and high-sensitivity C-reactive protein (hs-CRP) using an enzyme-linked immunosorbent assay (ELISA). Measure blood glucose, total cholesterol (TC), triglycerides (TG), low-density lipoprotein (LDL), high-density lipoprotein (HDL), blood urea nitrogen (BUN), and serum iron (Fe) using a fully automated biochemical analyzer. RDW, hemoglobin (Hb), and mean corpuscular volume (MCV) were measured using immunoturbidimetry; homocysteine (Hcy) was detected using the cyclic enzyme method.

### 2.6 Classification criteria

NOAF was defined as atrial fibrillation first occurring at admission or during hospitalization, detected via continuous telemetry monitoring for at least 72 h and confirmed by 12-lead ECG. Asymptomatic or paroxysmal episodes may have been under-detected due to the intermittent nature of ECG recording, which represents a limitation of this retrospective design. According to the 2023 American College of Cardiology/American Heart Association/Heart Rhythm Society guidelines for atrial fibrillation management (17), *P* waves are absent and replaced by irregularly sized and shaped f waves, most prominent in leads V1, II, III, and VF; atrial rate ranges from 350 to 600 beats per minute; and R-R intervals are absolutely irregular.

### 2.7 Statistical analysis

Data statistical analysis and visualization were performed using SPSS 26.0 and R 4.3.2 software. For normally distributed quantitative data, independent samples t-tests were applied, expressed as (x̅ ± s); non-normally distributed data were analyzed using the Mann–Whitney U test, expressed as M(P25,P75). Categorical variables were analyzed using chi-square tests. A significance level of *P* < 0.05 was adopted. A total of 26 candidate variables, comprising all demographic, clinical, and laboratory parameters listed in Tables 1, 2, were included in the initial variable pool for LASSO regression. LASSO regression with 10-fold cross-validation was employed for feature selection, using the optimal penalty parameter (*λ*) that minimized the mean squared error. The criteria for variable selection was the retention of variables with non-zero coefficients at this optimal *λ*. This process resulted in the selection of six predictors: gender, hospitalization duration, Killip class, NT-proBNP, RDW, and Hcy. Although we assessed linearity for continuous variables using restricted cubic splines, we ultimately adopted a standard logistic regression (linear) modeling framework for several reasons. First, given the limited number of outcome events (*n* = 41) and the low events-per-variable ratio (EPV = 6.8), adopting more complex nonlinear models (e.g., spline-based logistic regression or machine learning approaches) would substantially increase model complexity and the risk of severe overfitting, potentially yielding unstable and non-generalizable estimates. Second, our primary objective was an exploratory analysis of risk factor associations, for which standard logistic regression provides directly interpretable odds ratios that are clinically meaningful. Third, for the purpose of constructing a simple, user-friendly nomogram for visual hypothesis generation, a linear modeling framework offers superior transparency and ease of application compared to more complex nonlinear alternatives. Given the exploratory nature of this study and the limited number of outcome events (*n* = 41), the events-per-variable (EPV) ratio for the final multivariable logistic regression model containing these six predictors was approximately 6.8 (41/6), which is below the conventional threshold of 10. This low EPV increases the risk of model overfitting and necessitates cautious interpretation of the results. To assess the potential magnitude of overfitting, we performed internal validation using 1,000 bootstrap resamples to obtain an optimism-corrected estimate of model performance, including calculation of the optimism-corrected AUC and evaluation of coefficient shrinkage. The predictive value of individual factors was also assessed using the area under the curve (AUC). A nomogram was developed based on the logistic regression model using the rms package to visualize the identified risk factors. Calibration was assessed visually, and clinical utility was explored using decision curve analysis, with the understanding that these results are preliminary and require external validation.

**Table 1:** Comparison of baseline data and clinical characteristics of elderly AMI patients.

Indicator		NOAF Group (*n* = 41)	Non-noaf group (*n* = 163)	*x²/t/Z*	*P*
Age (years, *x̅* *±* *s*)		73.16 ± 8.33	73.27 ± 8.47	0.075	0.941
Gender (n)	male	23	127	8.011	0.005
	female	18	36		
Body Mass Index (kg/m², *x̅* *±* *s*)		24.16 ± 3.16	24.41 ± 3.31	0.436	0.663
Marital status (n)	Married	32	133	0.266	0.606
	Others	9	30		
Educational attainment (n)	Junior high school and below	24	91	0.098	0.755
	High school and above	17	72		
History of smoking (n)	have	23	58	5.759	0.016
	no	18	105		
History of alcohol consumption (n)	have	18	55	1.472	0.225
	no	23	108		
Combined with diabetes (n)	have	12	28	3.038	0.081
	no	29	135		
Combined with hypertension (n)	have	17	43	3.590	0.058
	no	24	120		
Heart rate at admission (Times/min, *x̅* *±* *s*)		82.31 ± 13.72	81.15 ± 11.68	0.548	0.584
Hospital stay [d, *M* (*P25*, *P75*)]		11.00 (6.00, 20.00)	9.00 (5.00, 12.00)	2.523	0.012
Systolic blood pressure (mmHg, *x̅* *±* *s*)		130.50 ± 16.87	127.56 ± 16.23	1.029	0.305
Diastolic blood pressure (mmHg, *x̅* *±* *s*)		77.65 ± 8.63	79.32 ± 10.21	0.964	0.336
Infarction site (n)	Front wall	18	74	0.142	0.986
	Lower wall	10	42		
	Composite wall	7	26		
	Others	6	21		
Number of diseased branches (n)	1 stick	16	81	1.495	0.221
	>1tick	25	82		
Killip classification of cardiac function (n)	Ⅰ Level	22	123	7.575	0.006
	Ⅱ∼ⅣLevel	19	40		
Target blood vessel（n)	Left main trunk	3	9	0.353	0.950
	Anterior descending branch	16	66		
	Spiral Branch	9	32		
	Right coronary artery	13	56		

**Table 2:** Comparison of laboratory indicators in elderly AMI patients.

Indicator	NOAF Group (*n* = 41)	Non-noaf group (*n* = 163)	*t/Z*	*P*
IL-6 (ng/L, *x̅* *±* *s*)	1.95 ± 0.57	1.72 ± 0.72	1.900	0.059
hs-CRP (mg/L, *x̅* *±* *s*)	6.21 ± 1.12	5.88 ± 1.04	1.788	0.075
TC (mmol/L, *x̅* *±* *s*)	4.42 ± 0.69	4.46 ± 0.71	0.324	0.746
TG (mmol/L, *x̅* *±* *s*)	1.43 ± 0.24	1.46 ± 0.21	0.794	0.428
LDL (mmol/L, *x̅* *±* *s*)	1.26 ± 0.34	1.19 ± 0.29	1.333	0.184
HDL (mmol/L, *x̅* *±* *s*)	2.89 ± 0.53	2.79 ± 0.45	1.226	0.222
BUN (mmol/L^−1^, *x̅* *±* *s*)	6.41 ± 2.87	6.02 ± 2.13	0.972	0.332
Fe (g/L^−1^, *x̅* *±* *s*)	4.25 ± 1.03	4.26 ± 1.23	0.048	0.962
Hb (g/L, *x̅* *±* *s*)	128.69 ± 19.13	131.88 ± 16.17	1.087	0.278
MCV (L, *x̅* *±* *s*)	92.25 ± 4.20	91.13 ± 6.14	1.104	0.271
NT-proBNP [ng/mL, *M* (*P25*, *P75*)]	2.08 (1.88, 2.58)	1.71 (1.42, 2.04)	4.607	<0.001
RDW (%, *x̅* *±* *s*)	15.16 ± 3.12	11.43 ± 4.14	5.393	<0.001
Hcy (µmol/L, *x̅* *±* *s*)	22.15 ± 1.83	19.63 ± 2.51	6.033	<0.001

Regarding the assessment of nonlinearity, our preliminary evaluation using restricted cubic splines suggested that the relationships between continuous predictors (NT-proBNP, RDW, Hcy) and the log-odds of NOAF were approximately linear within the observed data range, with no strong statistical evidence of significant nonlinear deviations (*P* for nonlinearity >0.05 for all). Therefore, we considered the linearity assumption to be reasonable for this exploratory analysis. Alternative modeling strategies such as spline-based logistic regression were considered but not ultimately selected, as they would introduce additional parameters and increase model complexity, which is particularly problematic given our small sample size and low event count. More complex machine learning approaches (e.g., random forests, support vector machines) were deemed unsuitable for this exploratory risk factor study due to their “black-box” nature and limited interpretability, which would conflict with our goal of identifying and visualizing interpretable risk associations. We acknowledge that future studies with larger sample sizes should revisit the potential nonlinear effects of these biomarkers using more flexible modeling techniques.

## 3 Results

### 3.1 Comparison of baseline data and clinical characteristics in elderly AMI patients

There were no statistically significant differences between the NOAF and non-NOAF groups in general demographic characteristics such as age and body mass index, nor in clinical indicators including heart rate, systolic blood pressure, and diastolic blood pressure at admission (*P* > 0.05). However, significant differences were observed between the two groups in gender distribution, smoking status, length of hospitalization, and Killip classification of cardiac function (*P* < 0.05), as shown in Table 1.

### 3.2 Comparison of laboratory indicators in elderly AMI patients

There were no statistically significant differences between the NOAF and non-NOAF groups in IL-6, hs-CRP, TC, TG, LDL, HDL, BUN, Fe, Hb, or MCV (*P* > 0.05). However, the NOAF group showed significantly higher levels of NT-proBNP ([2.08 (1.88, 2.58) vs. 1.71 (1.42, 2.04)], RDW (15.16 ± 3.12 vs. 11.43 ± 4.14), and Hcy (22.15 ± 1.83 vs. 19.63 ± 2.51) levels were significantly higher in the NOAF group than in the non-NOAF group (*P* < 0.05). See Table 2.

### 3.3 Analysis of Key variables for NOAF in elderly AMI patients

**Figure 1:**
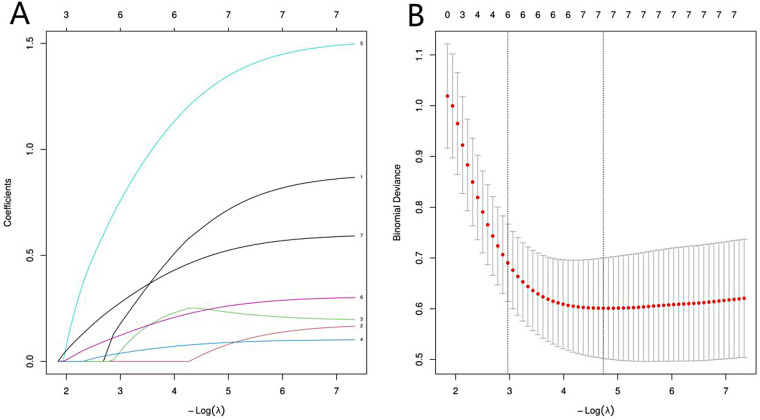
LASSO regression analysis. The lower axis of **(A)** displays the logarithmic value of log(*λ*), while the upper axis shows the number of retained variables. The vertical axis presents the standardized coefficients of each feature, with their variation trajectories visually represented by colored curves. As *λ* decreases (moving left on the *x*-axis), more variables enter the model. The order in which variables first enter the model (i.e., the order of importance based on LASSO selection) can be inferred from the sequence in which their coefficient trajectories depart from zero. In this analysis, the first variable to enter was Hcy, followed by RDW, NT-proBNP, Killip class, hospitalization duration, and finally gender, suggesting the relative strength of their associations with NOAF in the penalized selection process. **(B)**: The vertical axis represents mean squared error (MSE). The red vertical line indicates lambda.min, denoting the optimal variable subset corresponding to the minimum error-the number of independent variables yielding the smallest MSE. The black vertical line, lambda.lse, represents the simplified model selection where the error increases by one standard error.

To optimize model performance and address multicollinearity, LASSO regression was employed for feature selection among preliminarily screened differential variables (Figure 1A), compressing redundant regression coefficients to zero. To validate the reliability of the LassoLogit results, cvlassologit cross-validation was used to determine the optimal *λ* (Figure 1B). The *λ* value yielding the smallest error in 10-fold cross-validation (*λ* = 0.052) was ultimately selected as the optimal fitting parameter. Six variables were incorporated into the final model: gender, hospitalization duration, Killip class of cardiac function, NT-proBNP, RDW, and homocysteine (Hcy) level.

### 3.4 Logistic regression analysis of NOAF in elderly AMI patients

Variables selected by Lasso regression were incorporated into logistic analysis. Results indicated that gender, hospitalization duration, Killip classification of cardiac function, NT-proBNP, RDW, and Hcy were all risk factors for NOAF in elderly AMI patients (OR = 3.367, 1.110, 3.171, 4.677, 1.358, 1.814, *P* < 0.05). Model validation confirmed the original hypothesis: the model fit aligns with observed values (*χ*² = 6.957, *P* = 0.541 > 0.05), indicating meaningful model construction (see Table 3). Multicollinearity among predictors was assessed using variance inflation factors (VIFs), all of which were <3, indicating acceptable collinearity. The strongest correlation was observed between NT-proBNP and Killip class (*r* = 0.62), but VIF remained within acceptable limits.

**Table 3:** Logistic regression analysis of NOAF in elderly AMI patients.

Indicator	Regression coefficient	Standard error	*Z* value	Wald*χ*2	*P* value	OR value	OR value95%CI
Gender	1.214	0.529	2.294	5.262	0.022	3.367	1.193–9.498
Hospital stay	0.104	0.042	2.456	6.030	0.014	1.110	1.021–1.206
Killip classification	1.154	0.514	2.244	5.037	0.025	3.171	1.157–8.689
NT-proBNP	1.543	0.495	3.119	9.728	0.002	4.677	1.774–12.328
RDW	0.306	0.079	3.869	14.968	0.000	1.358	1.163–1.585
Hcy	0.596	0.127	4.681	21.916	0.000	1.814	1.414–2.328

### 3.5 Development of a risk prediction model for Non-orthostatic atrial fibrillation in elderly patients with acute myocardial infarction

**Figure 2:**
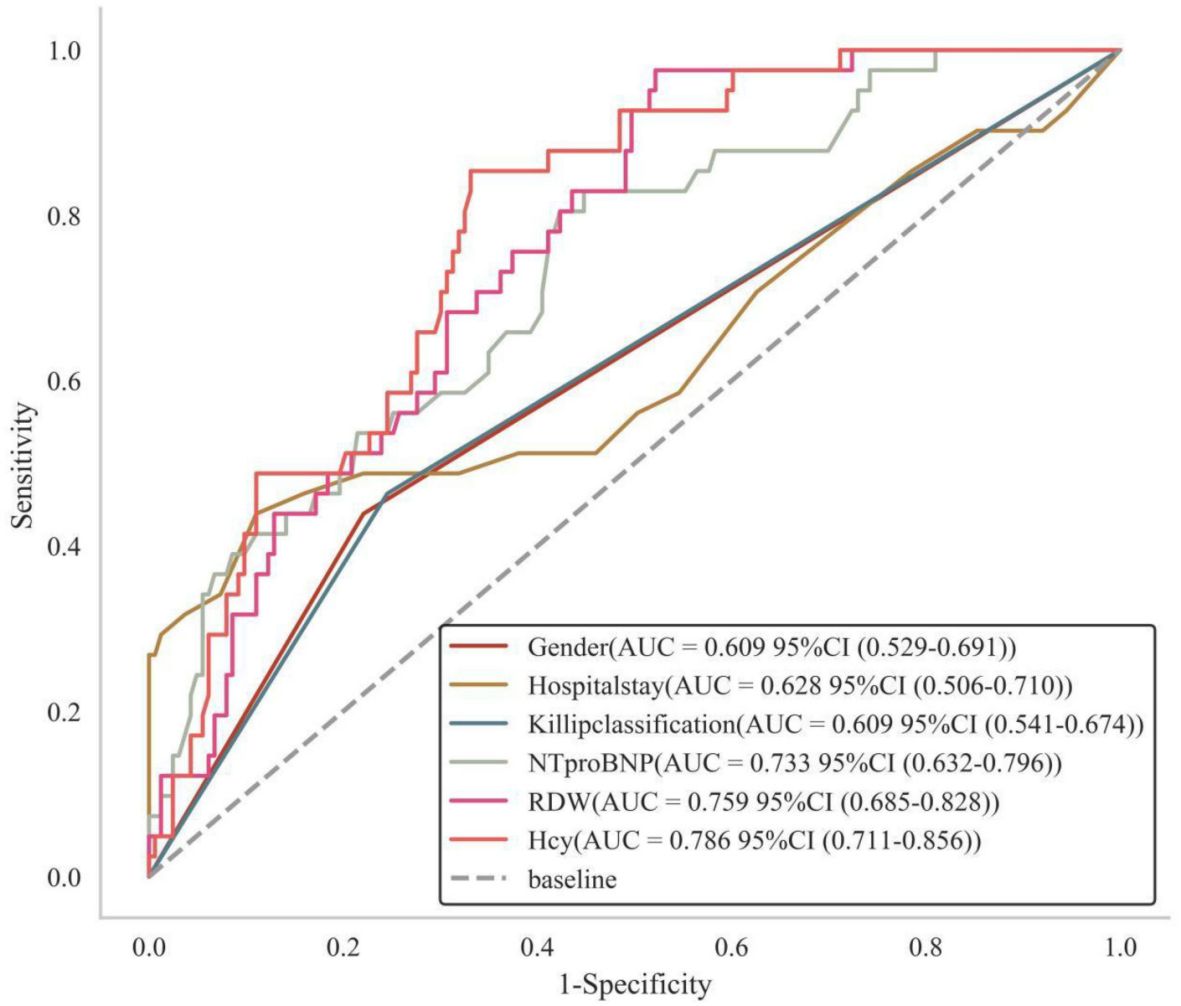
Shows the predictive value of each indicator for the occurrence of NOAF in elderly AMI patients.

**Figure 3:**
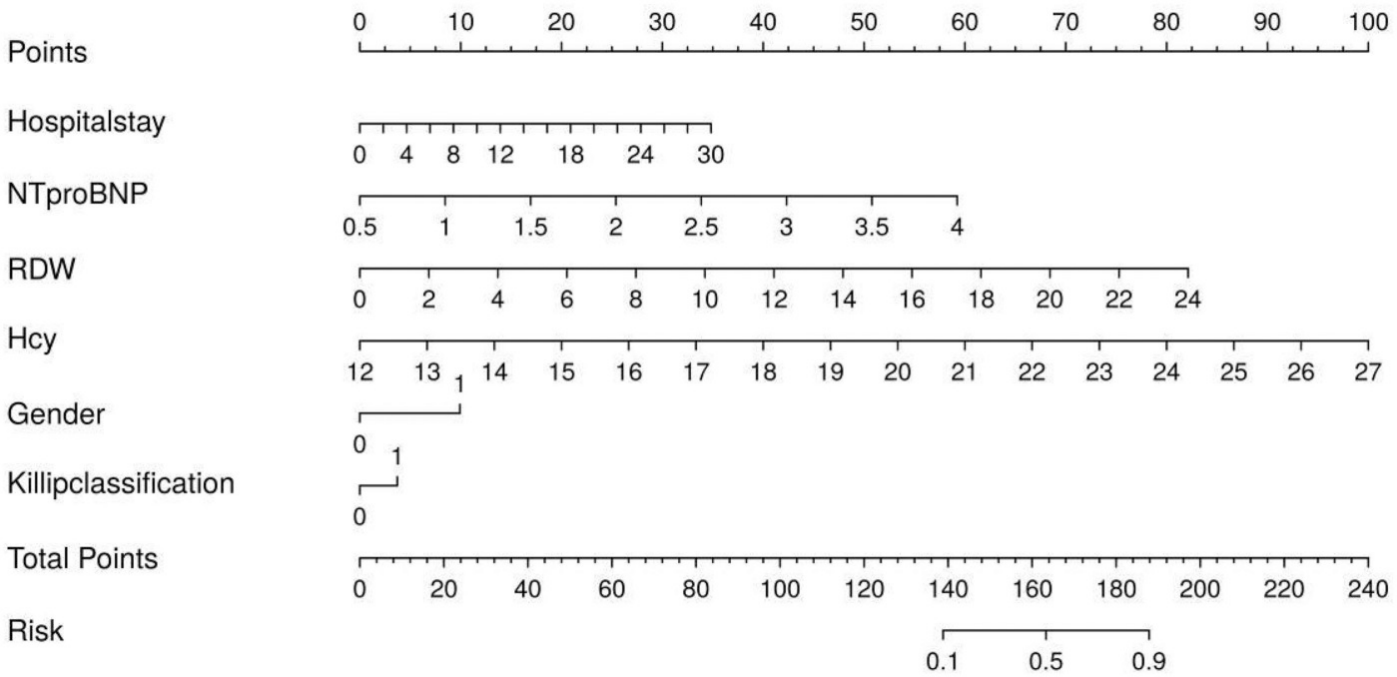
Nomogram of risk prediction for NOAF in elderly AMI patients.

ROC analysis revealed AUC values of 0.609, 0.628, 0.609, 0.733, 0.759, and 0.786 for gender, hospitalization duration, Killip class, NT-proBNP, RDW, and Hcy, respectively, in univariate analysis (Figure 2). The following logistic regression model was established based on the selected variables: Logit(p) = −22.569 + 1.214*Gender + 0.104*Hospitalization Duration + 1.154*Killip Class + 1.543*NT-proBNP +0.306*RDW + 0.596*Hcy. This model yielded an apparent AUC of 0.926 (95% CI: 0.880–0.973) in the development set. However, given the low EPV (6.8), this apparent performance is likely optimistic due to overfitting. After internal validation with 1,000 bootstrap resamples, the optimism-corrected AUC was 0.901. The difference of 0.025 between the apparent and optimism-corrected AUCs provides a quantitative estimate of the optimism bias inherent to this small dataset. While the corrected value of 0.901 still suggests good discriminative ability within this cohort, it is critical to emphasize that internal validation does not guarantee generalizability, and this finding must be interpreted with caution as it does not replace external validation in independent populations. A nomogram visualizing these factors was constructed (Figure 3).

### 3.6 Model performance evaluation

**Figure 4:**
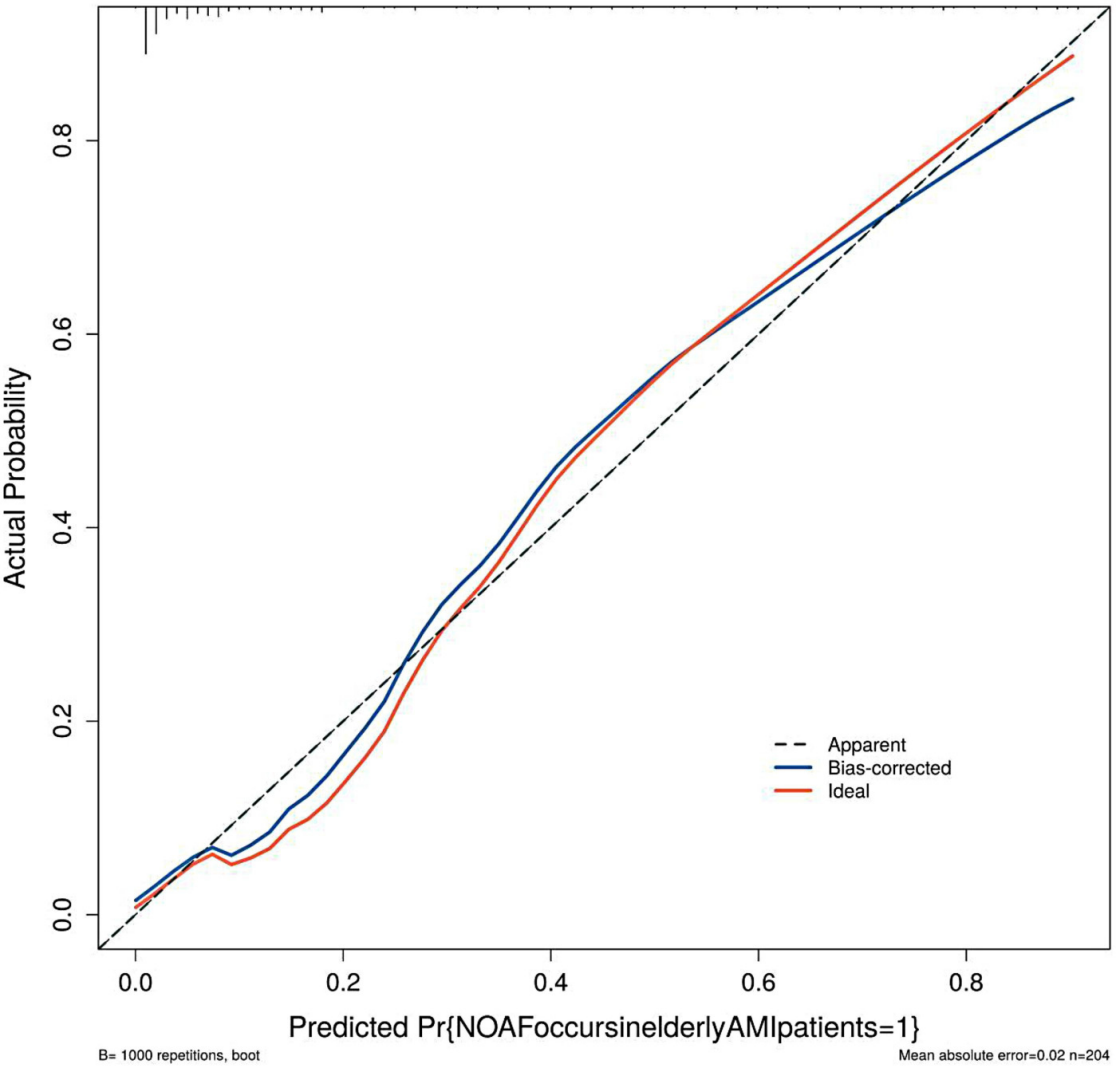
Verification of the calibration curve of the nomogram model.

**Figure 5:**
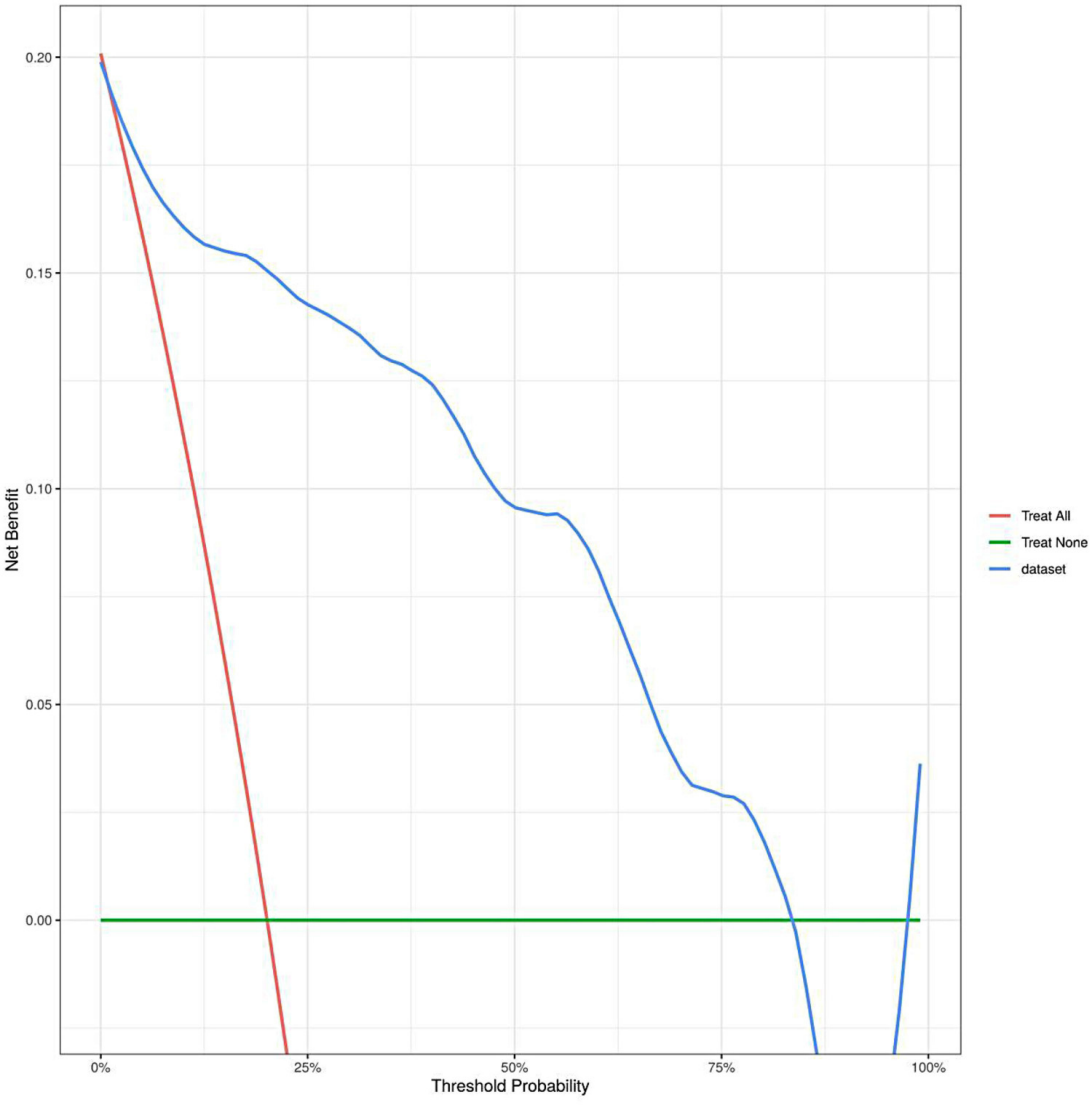
Verification of the decision curve of the nomogram model.

The model was validated using 1,000 bootstrap resamples, yielding an optimism-corrected AUC of 0.901, Cox-Snell R² of 0.593, and Nagelkerke R² = 0.376. Although the apparent AUC was 0.926, the optimism-corrected value of 0.901 indicates a reduction of 0.025 due to overfitting, underscoring the need for external validation. The calibration curve (Figure 4) visually assesses the agreement between predicted probabilities and observed outcomes. In this plot, the ideal 45-degree dashed line represents perfect calibration, where predicted probabilities exactly match observed frequencies. The apparent calibration curve (solid line) shows the model's fit to the development data, while the bias-corrected curve (blue line), derived from 1,000 bootstrap resamples, provides a more accurate estimate of calibration adjusted for overfitting. The close alignment of the bias-corrected curve with the ideal line suggests good calibration across the risk spectrum. The Hosmer-Lemeshow test (*χ*² = 6.957, *P* = 0.541) further supports good calibration, though this test has limitations in small samples. The decision curve analysis (Figure 5) evaluates the clinical utility of the model by plotting net benefit against various threshold probabilities. The *x*-axis represents the threshold probability at which a clinician would consider intervention, and the *y*-axis shows the net benefit relative to strategies of treating all or no patients. The curve for our nomogram (red line) lies above both the “treat all” (gray line) and “treat none” (horizontal line at zero) strategies across a clinically relevant threshold probability range of approximately 5% to 50%, suggesting that using the model to guide clinical decisions would yield higher net benefit than default strategies within this range. However, given the preliminary nature of this exploratory model, these findings require validation before informing clinical practice.

## 4 Discussion

In recent years, awareness of the hazards of atrial fibrillation (AF) has deepened. AF not only increases the incidence of stroke in elderly patients but also elevates the prevalence of cardiovascular disease, prolongs long-term mortality, and extends hospital stays (18, 19). Some AMI patients present with hemodynamic dysfunction. The presence of NOAF may further exacerbate this dysfunction, independently correlating with mortality in AMI patients. Moreover, NOAF patients exhibit higher rates of recurrent myocardial infarction and cerebral infarction, contributing to increased mortality (20, 21). Therefore, identifying risk factors for NOAF in AMI patients and implementing timely interventions are crucial for improving patient outcomes.

The occurrence of NOAF is directly associated with myocardial tissue injury. Our exploratory study identified NT-proBNP, RDW, and Hcy as factors significantly associated with NOAF in elderly AMI patients. NT-proBNP, a marker of myocardial stretch and volume overload (22, 23), reflects the hemodynamic burden imposed by AMI. RDW, increasingly recognized as an integrative marker of systemic inflammation and oxidative stress (24, 25), captures the inflammatory milieu that promotes atrial electrical and structural remodeling. Hcy contributes to endothelial dysfunction and prothrombotic states (26, 27), further destabilizing the atrial substrate. The convergence of these biomarkers-representing hemodynamic stress, inflammation, and endothelial dysfunction-underscores the multifactorial pathogenesis of NOAF in the post-AMI setting. This aligns with contemporary evidence highlighting the complex interplay of atrial substrate remodeling, autonomic dysregulation, and systemic inflammatory activation as key determinants of AF (doi: 10.1111/jce.70128). Our findings extend this perspective by demonstrating that integrating these distinct yet interconnected pathways within a single model may offer a more comprehensive assessment of NOAF risk than any single parameter alone.

Data from this study indicate significant differences between the NOAF and non-NOAF groups in gender, smoking status, length of hospitalization, and Killip class. Furthermore, in the multivariable analysis, gender, hospitalization duration, Killip class, NT-proBNP, RDW, and Hcy showed significant associations with NOAF. The association with female gender may be attributed to distinct patterns of myocardial ischemia that disrupt cardiac electrical stability (28). The association with Killip class, a marker of heart failure severity, aligns with the well-established link between volume overload, atrial stretch, and arrhythmogenesis (29). However, the inclusion of “hospitalization duration” requires careful interpretation. While significantly associated with NOAF in our model, it is likely a downstream consequence of the event itself or its complications, rather than a true antecedent risk factor. Therefore, it should be considered a marker of illness severity or a surrogate for unmeasured confounders (e.g., in-hospital complications, electrolyte imbalances), not a causal predictor for baseline risk stratification. Its inclusion in the nomogram is for exploratory visualization of this association, but it limits the model's utility for early prediction upon admission.

Smoking history, while significant in univariate analysis, was not retained in the LASSO-Logistic model. This could be due to several factors inherent to the exploratory LASSO procedure. First, smoking's pro-arrhythmic effects are largely mediated through chronic inflammatory and oxidative stress pathways, which may be more proximally captured by other retained variables like RDW. Second, in the context of an acute event like AMI, the direct and immediate effects of myocardial necrosis (reflected by NT-proBNP and Killip class) may dominate the statistical signal, overshadowing the longer-term contribution of smoking. Third, LASSO regression, by design, performs automated feature selection to create a parsimonious model, potentially excluding a correlated variable with a weaker independent signal. This finding does not negate the biological importance of smoking in cardiovascular disease but suggests that within this specific, small dataset focused on an acute complication, its statistical contribution was not independent of the other, more powerful, clinical and biomarker signals. This remains a hypothesis-generating observation for future research (30, 31). Previous studies indicate that nomograms can transform complex regression equations into graphical representations. In this exploratory study, a nomogram was constructed to visualize the multivariate associations (Figure 3). To illustrate its potential application if validated in future studies, the nomogram can be used as follows: each predictor value corresponds to a point score on the top scale. For example, a female patient (approximately 18 points) with a Killip class II-IV (approximately 17 points), an NT-proBNP level of 2.5 ng/mL (approximately 23 points), an RDW of 16% (approximately 20 points), and an Hcy of 23 µmol/L (approximately 24 points), with a hospitalization duration of 14 days (approximately 12 points), would accumulate a total score of approximately 114 points. Projecting this total score vertically onto the bottom probability scale yields an estimated risk of NOAF exceeding 70%. While this example demonstrates the practical mechanics of the nomogram, we strongly caution that due to the aforementioned methodological limitations-particularly the low EPV, inclusion of the temporally ambiguous variable “hospitalization duration,” and lack of external validation-this tool is not ready for clinical application. The nomogram is presented here not as a validated clinical tool, but as a visual summary of the exploratory model's associations to facilitate hypothesis generation and inform the design of future confirmatory studies. Calibration curves demonstrated reasonable agreement between model predictions and observed NOAF events within this dataset, though this assessment is subject to the same overfitting concerns. The clinical decision curve analysis suggested a potential net benefit within a range of threshold probabilities, but these performance metrics are preliminary and likely optimistic.

Limitations of this exploratory study include: ① The modest sample size and low number of events (*n* = 41) resulted in an EPV of approximately 6.8, which is below the recommended threshold for stable predictive modeling, increasing the risk of overfitting. ② A key methodological limitation is that feature selection using LASSO was performed on the entire dataset (with internal cross-validation), and the same data were subsequently used for model evaluation. Although we applied bootstrap resampling for internal validation, this approach does not fully eliminate the optimistic bias inherent in using the same dataset for both feature selection and model performance assessment. The optimism-corrected AUC of 0.901 should therefore be interpreted with caution, as it may still overestimate true performance in independent populations. Truly unbiased performance estimation would require a completely independent external validation cohort or a nested cross-validation framework where feature selection is repeated within each bootstrap iteration, which was not feasible given our sample size constraints. ③ The inclusion of hospitalization duration, a variable that occurs concurrently with or after the outcome, limits the model's utility for true prediction and introduces temporal ambiguity. ④ Lack of external validation means model performance cannot be generalized. ⑤ Data on key medications (e.g., beta-blockers, antiarrhythmics) and revascularization strategies, which could influence NOAF risk, were unavailable. ⑥ Short follow-up may underestimate NOAF incidence. Consequently, the findings of this study should be viewed as hypothesis-generating. The identified factors require confirmation in larger, prospective cohorts designed with clear temporal separation between predictor assessment and outcome occurrence. Future research should also aim to include a more comprehensive set of clinically relevant variables and employ more rigorous validation frameworks.

Previous studies indicate that nomograms transform complex multivariate regression equations into graphical representations, rendering abstract data outcomes visualizable and readable to achieve effective prediction and assist clinicians in decision-making (32). In this study, a nomogram prediction model was constructed by integrating multiple factors to assess the approximate probability of NOAF occurrence in elderly AMI patients using different predictive variables. Calibration curves demonstrated good agreement between model predictions and actual NOAF events in patients, This indicates high consistency between model predictions and observed outcomes, further enhancing the model's credibility. The clinical decision curve generally lies above both extreme curves, demonstrating that the factors included in the nomogram provide substantial net benefit in predicting NOAF in elderly AMI patients, thereby offering robust support for clinical decision-making. Limitations of this study include: ① Short follow-up duration may underestimate NOAF incidence. ② Lack of data on medications (e.g., beta-blockers, ACEI/ARB, antiarrhythmics) and revascularization details, which could influence NOAF risk. ③ The model lacks external validation. ④ The term “non-orthostatic arrhythmia” has been corrected to “new-onset atrial fibrillation” throughout. ⑤ Language has been edited for clarity and consistency. ⑥ Speculation on smoking exclusion has been tempered; future studies should examine its role in NOAF pathogenesis. While the model demonstrates high value in identifying high-risk patients, specific predicted probability values require cautious interpretation or calibration in external real-world cohorts. Future research should address these limitations to enhance the accuracy and practicality of predictive models for NOAF risk in elderly AMI patients.

## 5 Conclusion

In summary, this exploratory study identifies gender, hospitalization duration, Killip class, NT-proBNP, RDW, and Hcy as factors significantly associated with NOAF in elderly AMI patients within this retrospective cohort. The constructed nomogram visualizes these associations. However, due to significant methodological limitations-including a small sample size, low events-per-variable ratio, and the inclusion of a temporally ambiguous variable (hospitalization duration)-these findings should be considered preliminary and hypothesis-generating. The model is not suitable for clinical use. Further rigorous validation in large, prospective cohorts is essential before any potential clinical application can be considered.

## Data Availability

The original contributions presented in the study are included in the article/Supplementary Material, further inquiries can be directed to the corresponding author/s.
